# Predicting Human Age with Bloodstains by sjTREC Quantification

**DOI:** 10.1371/journal.pone.0042412

**Published:** 2012-08-03

**Authors:** Xue-ling Ou, Jun Gao, Huan Wang, Hong-sheng Wang, Hui-ling Lu, Hong-yu Sun

**Affiliations:** 1 Department of Forensic Medicine, Zhongshan School of Medicine, Sun Yat-sen University, Guangzhou, Guangzhou, People’s Republic of China; 2 Reproductive Medicine Center, First Affiliated Hospital of Sun Yat-sen University, Guangzhou, Guangzhou, People’s Republic of China; 3 Department of gynaecology, Third Affiliated Hospital of Sun Yat-sen University, Guangzhou, Guangzhou, People’s Republic of China; 4 Department of Microbial and Biochemical Pharmacy, School of Pharmaceutical Sciences, Sun Yat-sen University, Guangzhou, People’s Republic of China; Sudbury Regional Hospital, Canada

## Abstract

The age-related decline of signal joint T-cell receptor rearrangement excision circles (sjTRECs) in human peripheral blood has been demonstrated in our previous study and other reports. Until now, only a few studies on sjTREC detection in bloodstain samples were reported, which were based on a small sample of subjects of a limited age range, although bloodstains are much more frequently encountered in forensic practice. In this present study, we adopted the sensitive Taqman real-time quantitative polymerase chain reaction (qPCR) method to perform sjTREC quantification in bloodstains from individuals ranging from 0–86 years old (*n* = 264). The results revealed that sjTREC contents in human bloodstains were declined in an age-dependent manner (*r* = −0.8712). The formula of age estimation was Age  = −7.1815*Y*−42.458±9.42 (*Y* dCt_TBP-sjTREC_; 9.42 standard error). Furthermore, we tested for the influence of short- or long- storage time by analyzing fresh and stored bloodstains from the same individuals. Remarkably, no statistically significant difference in sjTREC contents was found between the fresh and old DNA samples over a 4-week of storage time. However, significant loss (0.16–1.93 dCt) in sjTREC contents was detected after 1.5 years of storage in 31 samples. Moreover, preliminary sjTREC quantification from up to 20-year-old bloodstains showed that though the sjTREC contents were detectable in all samples and highly correlated with donor age, a time-dependent decrease in the correlation coefficient *r* was found, suggesting the predicting accuracy of this described assay would be deteriorated in aged samples. Our findings show that sjTREC quantification might be also suitable for age prediction in bloodstains, and future researches into the time-dependent or other potential impacts on sjTREC quantification might allow further improvement of the predicting accuracy.

## Introduction

Individual age is an important phenotypic trait in forensic practice. Until now, age estimation from biological materials depends mostly on morphological [1] or biochemical methods [2]. When forensic samples such as bloodstain samples have no enough morphologic or biochemical information, approaches based on molecular biology are expected to provide some useful information. However, previously proposed genetic indicators for human age estimation, including the mitochondrial DNA 4, 977 deletion accumulations [3] or telomere shortening [4], [5], are still in the early stage due to the interferences of the environmental, genetic and disease effects. It could be imagined that individual age is too complex to allow only a simple molecular indicator in age estimation from biological materials. Thus, complementary studies on newer age-related indicators are expected to improve the age predicting accuracy with the assistance of methods based on molecular biology.

The critical role of the thymus in the generation of a diversified population of peripheral T lymphocytes is well-established. Recently, signal joint T-cell receptor (TCR) rearrangement excision circle (sjTREC) becomes one of the new tools for measuring thymic export [6], though the interpretation of sjTREC is revealed to be also affected by processes such as the proliferation and death of T cells in further researches [7]. sjTRECs are extra-chromosomal DNA by-products of the rearrangements of gene segments encoding the variable parts of TCR á and â chains during intra-thymic development. In this process, the intervening DNA segments in the TCR loci are deleted and the circularized DNA molecules are formed, so-called sjTRECs. δRec-ψJα sjTREC is one particular sjTREC arising through an intermediate rearrangement in the TCRD/A locus in developing TCRαβ+ T lymphocytes [8].

**Table 1:** Number and age distribution of the subjects studied.

Age Group (Year)	Male	Female	Total
0–4	8	12	20
5–9	14	7	21
10–14	15	7	22
15–19	7	6	13
20–24	5	10	15
25–29	9	10	19
30–34	8	11	19
35–39	16	11	27
40–44	20	14	34
45–49	19	7	26
50–54	13	10	23
55–59	4	4	8
60–64	3	3	6
65–	5	6	11
Total	146	118	264

As shown in our previous study and other reports, there is a log-linear sjTREC decline in human peripheral blood with increasing age [9]–[14], echoing the age-related thymic adipose involution in a life-long process and consequent thymic function loss. In addition, Zubakov et al and our study had demonstrated the strong relationship between normalized sjTREC quantification and individual age in fresh blood samples, showing promise for forensic application [13], [14]. However, it’s not fresh blood but bloodstain which is much more frequently encountered in a crime scene. Some reports had suggested that sjTREC quantification could be replicated in dried bloodstains [13], [15], whereas most of these studies were based on a small sample at limited storage conditions. Therefore, in this present study, we adopted the sensitive and flexible Taqman real-time quantitative polymerase chain reaction (qPCR) method described previously [14], to perform sjTREC quantification in bloodstain samples from a large series (*n* = 264) of unrelated healthy individuals in various storage conditions, so as to clarify whether it could be also suitable for age estimating of dry bloodstain samples as well.

**Figure 1:**
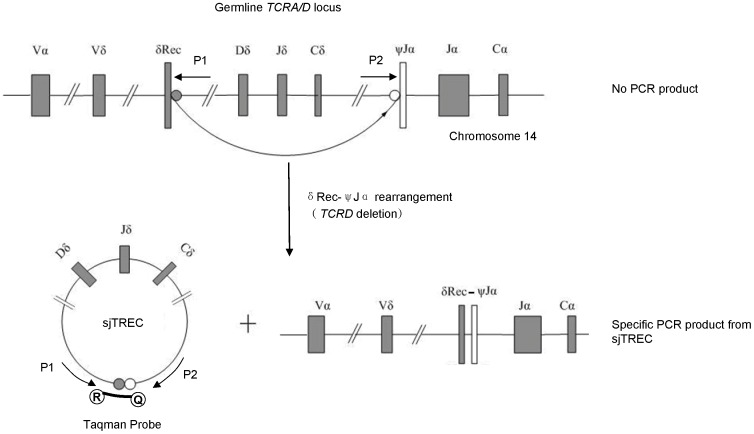
PCR amplification with P1/P2 primers. Using this primer set, only a TREC containing a unique signal joint (sj) sequence is amplified. The Taqman probe harbours an Reporter (R) in its 5¢ terminate and Quencher (Q) in 3¢ terminate.

## Materials and Methods

### Samples

Bloodstain samples were collected with cotton gauze from 264 unrelated healthy individuals respectively ranging from 0–86 years old of Chinese Han people, an ethnic group living in the southern China (Table 1). DNA extraction was then performed after the samples were dried overnight at lab conditions. Furthermore, to investigate the effect of long-time storage on assay performance, 31 of these 264 volunteers described above donated additional copies of bloodstains, which stored for 1.5 years until needed for DNA isolation. Besides, the studied old bloodstain samples which have been stored for 3 years (*n* = 20), 6 years (*n* = 19), 12 years (*n* = 18) and over 20 years (*n* = 20) were routinely kept at lab conditions for research purpose.

**Table 2:** sjTREC levels (dCt_TBP-sjTREC_) for five year cohorts of healthy individuals (males and females combined) with descriptive statistics for each cohort.

Age group	*n*	Minimum	Maximum	Mean±SE
0–4	20	−8.35	−5.26	−7.02±0.89
5–9	21	−8.78	−6.00	−7.56±0.73
10–14	22	−9.54	−5.64	−7.95±0.99
15–19	13	−10.63	−7.64	−9.08±0.83
20–24	15	−12.14	−8.52	−9.86±1.02
25–29	19	−12.13	−8.24	−10.12±0.98
30–34	19	−11.85	−9.21	−10.53±0.83
35–39	27	−13.01	−8.76	−10.91±1.15
40–44	34	−16.09	−9.45	−11.63±1.68
45–49	26	−13.87	−9.66	−11.67±1.27
50–54	23	−14.80	−9.64	−12.18±1.33
55–59	8	−15.62	−12.01	−13.67±1.15
60–64	6	−14.35	−11.14	−13.22±1.24
65–	11	−16.51	−13.24	−14.94±1.15

**Figure 2:**
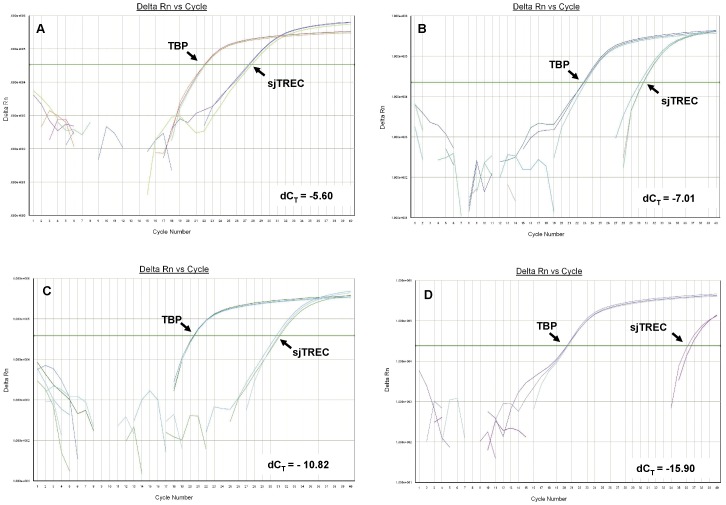
Typical illustrations of sjTREC and TBP amplification curves. Panel A to D was obtained for bloodstain samples from 0.1-, 11-, 31-, and 73-year-old individuals, respectively.

### Ethics Statement

The research protocol was approved by the Human Subjects Committee at the Zhongshan School of Medicine, Sun Yat-sen University. Written informed consent was attained by all participants or guardians involved in the study.

**Figure 3:**
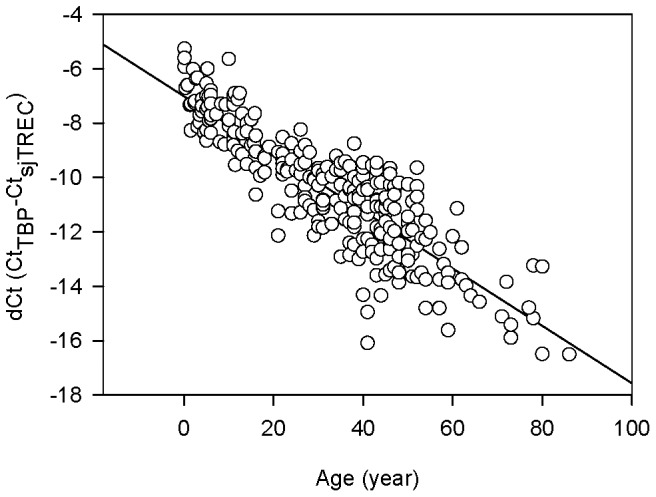
SjTREC levels in 264 bloodstain samples (aged 0–86 years old). sjTREC contents declined progressively in these collected samples with increasing donor age through a life span (*r* = −0.8712).

### DNA Isolation

DNA from the bloodstain samples was isolated using the Forensic DNA Kit® (Omega) according to the manufacturer’s instructions and stored at −20°C until use. Sample purity and quantity was determined by a Gene Quant pro RNA/DNA calculator (Amersham Pharmacia, USA).

**Figure 4:**
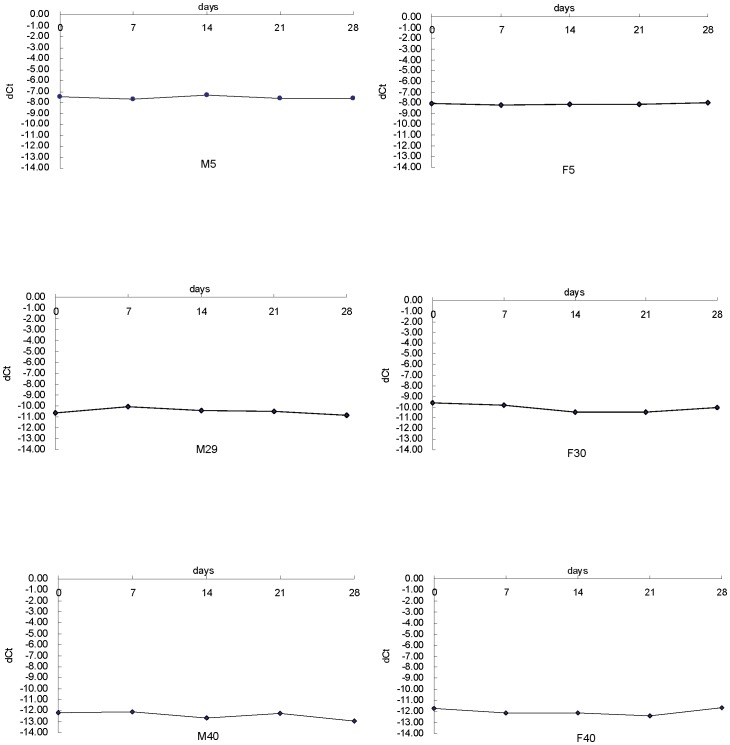
SjTREC levels of bloodstain samples quantified serially over a 28-day of storage time from 6 healthy volunteers. DNA was extracted every 7 days from the 6 involved volunteers and stored at −20°C until analysis. Gender and age for each individual was given.

### Taqman qPCR Assays

QPCR was performed using an ABI Prism® 7500 Sequence Detector System and the data was analyzed using SDS 2.0 software. Taqman™ Hydrolysis technology was used in a 20 µl reaction mixture containing 250 nM of each primer set, 250 nM Taqman hydrolysis probe and 10 µl Premix Ex Taq™ (TaKaRa). The sequence of primers and probe for sjTREC (ACCESSION AE000521) were 5′-CCATGCTGACACCTCTGGTT-3′ (P1: forward primer), 5′-TCGTGAGAACGGTGAATGAAG-3′ (P2: reverse primer), and 5′-FAM-CACGGTGATGCATAG GCACCTGC-TAMRA-3′ (Taqman probe) [16]. In addition, primers and probe for the internal reference gene, human TATA box binding protein (TBP; accession NG00816) were 5′-TTAGCTGGCTCTGAGTATGAATAAC-3′ (forward primer), 5′-AACCAATAAAACCTACTCC TCCCTTAA-3′ (reverse primer), and 5′- FAM-CAGTCCAGACTG GCAGCAAGAAAAT-TAMRA-3′ (Taqman probe). Schematic representation of the formation of sjTREC molecules during genomic DNA rearrangements in T-cells, along with the binding sites for the primers and probe of sjTREC TaqMan assay is presented at the Figure 1. Thermal cycling conditions were 95°C for 5 min then 95°C for 15 s, 60°C for 15 s, and 72°C for 32 s for 40 cycles. All reactions were performed in triplicates, and the average value of each sample was used for further data analysis. The normalized sjTREC amount in each sample was calculated as a difference between Ct values of TBP and sjTREC assays (dCt) as Zubakov et al. have described [13]. The efficiencies (*E*) of the PCR reactions were calculated by using the formula *E* = [10^(−1/*S*)^]−1 [17], with *S* being the slope of the standard curve.

**Figure 5:**
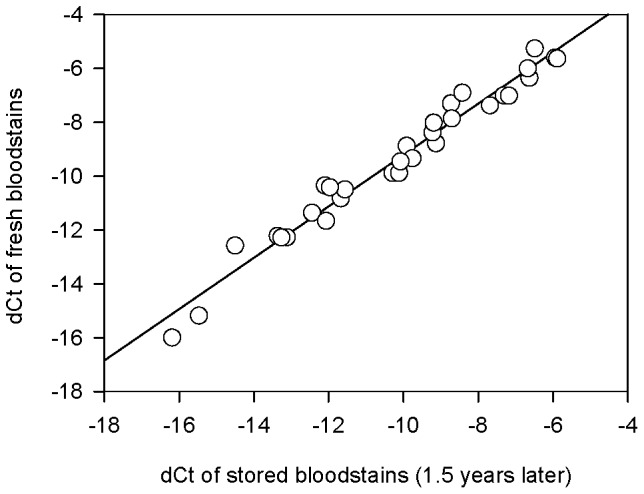
SjTREC levels in fresh vs stored bloodstain samples (31 pairs). Two copies of bloodstain samples were collected from a same donor, and then one copy was tested for sjTREC level immediately while another was stored for 1.5 years before quantification. Significant difference was observed in the sjTREC contents between fresh and stored samples (paired t test, p<0.01).

**Table 3:** sjTREC levels (dCt_TBP-sjTREC_) in bloodstain samples detected immediately (fresh bloodstains) or after storing at a room temperature for 1.5 years (stored bloodstains).

		Fresh bloodstains		Stored bloodstains (1.5 years)		
Case	Actual age (year)	dCt	Calculated age (year)	dCt	Calculated age (year)	dCt loss
1	0.1	−5.26	−4.68±9.42*	−6.49	4.13±9.42*	1.23
2	0.83	−5.60	−2.24±9.42*	−5.94	0.22±9.42*	0.34
3	2.7	−6.35	3.17±9.42*	−6.63	5.18±9.42*	0.28
4	5	−7.00	7.79±9.42*	−7.31	10.06±9.42*	0.32
5	5.2	−6.00	0.61±9.42*	−6.68	5.49±9.42*	0.68
6	8	−7.37	10.47±9.42*	−7.69	12.74±9.42*	0.32
7	8.3	−7.29	9.92±9.42*	−8.73	20.21±9.42**	1.43
8	9	−8.78	20.60±9.42**	−9.13	23.09±9.42**	0.35
9	10	−5.64	−1.98±9.42**	−5.89	−0.16±9.42**	0.25
10	11	−7.02	7.93±9.42*	−7.18	9.08±9.42*	0.16
11	11.3	−6.91	7.14±9.42*	−8.43	18.06±9.42*	1.52
12	13	−8.38	17.70±9.42*	−9.23	23.79±9.42**	0.85
13	14	−8.01	15.09±9.42*	−9.19	23.54±9.42**	1.18
14	15	−7.86	14.01±9.42*	−8.70	20.04±9.42*	0.84
15	20	−8.87	21.27±9.42*	−9.91	28.71±9.42*	1.04
16	22	−9.34	24.59±9.42*	−9.76	27.61±9.42*	0.42
17	22	−9.89	28.54±9.42*	−10.29	31.46±9.42**	0.41
18	31	−9.89	28.54±9.42*	−10.11	30.17±9.42*	0.23
19	31	−10.82	35.27±9.42*	−11.67	41.35±9.42**	0.85
20	38	−10.49	32.90±9.42*	−11.56	40.58±9.42*	1.07
21	40	−9.45	25.43±9.42**	−10.08	29.91±9.42**	0.62
22	41	−10.35	31.85±9.42*	−12.10	44.41±9.42*	1.75
23	41	−10.41	32.33±9.42*	−11.96	43.46±9.42*	1.55
24	41	−11.36	39.12±9.42*	−12.44	46.90±9.42*	1.08
25	41	−12.26	45.61±9.42*	−13.12	51.74±9.42**	0.85
26	50	−11.67	41.33±9.42**	−12.07	44.20±9.42*	0.40
27	53	−12.22	45.32±9.42*	−13.36	53.51±9.42*	1.14
28	54	−12.28	45.71±9.42*	−13.27	52.82±9.42*	0.99
29	62	−12.57	47.84±9.42**	−14.50	61.70±9.42*	1.93
30	73	−16.00	72.42±9.42*	−16.19	73.79±9.42*	0.19
31	78	−15.18	66.56±9.42**	−15.47	68.65±9.42*	0.29

* Inside the range of standard error (S. E.),

** Outside the range of S.E.

### Statistics

The normality of the data was examined using the Kolmogorov-Smirnov test, and homogeneity of variance using the Levene median test. Linear regression equation was obtained for estimating the age of donors by sjTREC levels in bloodstain samples. Gender difference was assessed using the unpaired student *t* test, and differences in dCt in bloodstains quantified over a serial of storage time using the paired *t* test. All statistical analysis was performed using SPSS^©^ 16th edition software. Results were considered significant when *p*<0.05.

**Figure 6:**
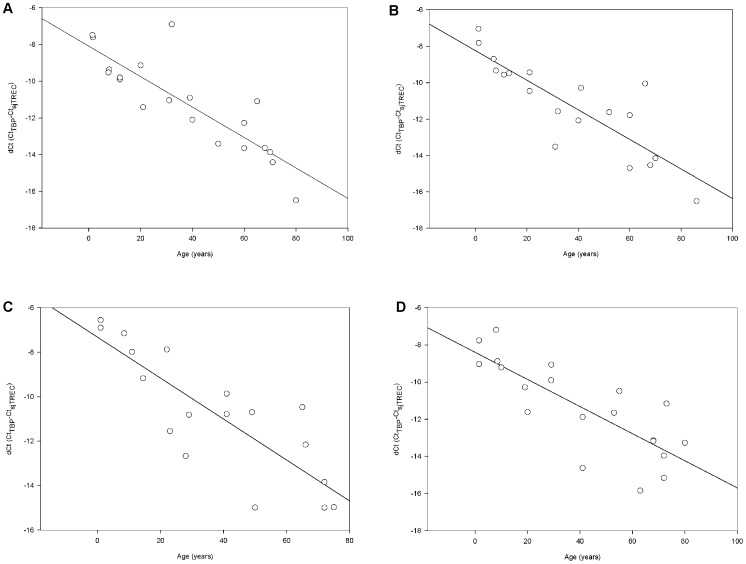
sjTREC quantification from up to 20-year-old bloodstains. SjTREC levels declined with increasing donor age in old bloodstain samples which had stored for A) 3 years (*r* = 0.8566, *n* = 20), B) 6 years (*r* = 0.8478, *n* = 19), C) 12 years (*r* = 0.8346, *n* = 18) and D) over 20 years (*r* = 0.7991, *n* = 19).

## Results

### Linearity, Amplification Efficiency, and Sensitivity of the qPCR Assay

Plasmid constructs encoding the sjTREC sequence or TBP were used as standards and diluted over the range 10^6^–10^1^. A linear range was observed for both the sjTREC and TBP amplicons, and their efficiencies were very close to each other (0.940±0.021 for sjTREC and 1.037±0.092 for TBP), which is a prerequisite to the use of direct dC_T_ method for accurate normalized sjTREC quantification [18]. Moreover, the lowest amount of sjTREC and TBP that could be reliably quantified both corresponded to 10 copies per reaction.

### Normalized sjTREC Quantification in Bloodstain Samples

The sjTREC quantification by qPCR analysis was successfully performed in all 264 collected bloodstain samples (Table 2). Typical illustrations of amplification curves and quantification results obtained for bloodstain DNA samples from 0.1-, 11-, 31-, and 73-year-old individuals were demonstrated in Figure 2 (from A to D). In contradistinction to the TBP normalizer, which was stable across all ages, sjTREC contents reduced as the donor age increased (Figure 2).

We then analyzed the normalized sjTREC levels (dCt_TBP-sjTREC_) in a cohort of 264 bloodstain samples attained from healthy subjects ranging from 0–86 years old. As illustrated in Figure 3, sjTREC contents declined progressively in these collected samples with increasing donor age through a life span, and it should be noted that even people over the age of 80 had significant numbers of sjTREC in their bloodstains.

### Correlation between Normalized sjTREC Quantification and Individual Age

Considering the donor age as the dependent variable and sjTREC levels (dCt values) as the predictor, we adopted linear regression to assess the correlation between sjTREC level and individual age. Linear regression equation was also obtained for age estimating, with its accuracy evaluated using *R* ^2^. A straight line was obtained by regression analysis between individual age and dCt, *R^2^* being 0.759 (*r* = −0.8712) (Figure 3). The normalized sjTREC level of each sample could be included in the following formula to predict the donor age: Age  = −7.1815*Y*–42.458±9.42 (*Y* dCt_TBP-sjTREC_; 9.42 standard error).

### Assessment of Gender Difference in sjTREC Quantification

Normalized sjTREC contents in male and female samples were compared to find out whether there is gender-specific change with regard to sjTREC level in human bloodstains. Not surprisingly, no differences were found between males and females in all 5-year groups (data not showed), which was concordant with our previous study on fresh whole blood samples [14].

### Stability of sjTREC Levels Over a 28-day Period of Time in Bloodstain Samples

Aiming to assure successful amplification of degraded DNA, we chose small amplicon sizes of 132 bp for sjTREC and 112 bp for TBP, and then sought to examine the stability of sjTREC quantification in bloodstain samples within the same individual, which have been stored for 0, 7, 14, 21, 28 days respectively. Six healthy male and female volunteers had their sjTREC in bloodstain samples quantified serially over a 28-day of storage time. As showed in Figure 4, fresh and old DNA samples over this period demonstrated very high similarity of dCt values and no significant difference was found (paired t test, p>0.05), suggesting that sample storage time within 4 weeks exerts no significant influence on sjTREC quantification.

### Influence of Sample Long-time Storage

Thirty-one volunteers were involved in this analysis. Two copies of bloodstain samples were collected with cotton gauze from a same donor, and then one copy was tested for sjTREC level immediately while another was stored for 1.5 years before sjTREC quantification. Notably, significant difference was observed in the sjTREC contents between fresh and stored samples (paired *t* test, p<0.01), with 0.16–1.93 dCt loss after a storage time of 1.5 years (Figure 5, Table 3). Moreover, the calculated age of only 22 of these 31 stored samples (70.95%) was inside the range of SE, whereas the accuracy of those fresh bloodstain samples was about 80.65%.

### Preliminary sjTREC Quantification from up to 20-year-old Bloodstains

Preliminary sjTREC quantification in old bloodstains was performed using samples routinely stored for 3, 6, 12 and over 20 years in lab condition. As demonstrated in Figure 6, the quantification was successfully in all collected bloodstains stored for over 3 years. A gradual individual age dependent decline in sjTREC contents was also seen, with a decrease in the correlation coefficient *r* along with the storage time (Figure 6). Furthermore, it should be noticed that even people at age of 80 had significant detectable sjTREC numbers in his bloodstains stored for over 20 years.

## Discussion

Besides routinely used morphologic indicators, a variety of biological features have been proved to be closely related to individual age and useful in age determination with forensic samples that cannot provide enough morphologic information. However, the practical value of these biological indicators seems limited for various reasons, and newer age-related markers will be expected to complementary for the current techniques for age estimation. Previously, we have reported a log-linear decline in peripheral sjTREC number with increasing human age in a life-long process, suggesting that assessment of sjTREC in peripheral blood might be a valuable additional tool in age determination [14]. However, only a few studies on sjTREC detection in bloodstain samples were reported, based on a small sample of subjects, although bloodstains are much more common in a crime scene.

In the present study, we employed the Taqman-based qPCR approach to quantify sjTREC levels, normalized to the single-copy TBP gene to account for the amount of input DNA, in bloodstain samples of 264 healthy Chinese individuals ranging from 0–86 years old. In contradistinction to using SYBR Green I dye, which detects all amplified double-stranded DNA including non-specific reaction products, the Taqman-based assay employs a fluorogenic probe to enable the detection of specific amplification products only and is widely used in sjTREC quantification in recent researches [12], [13], [15], [16]. The quantification results (Figure 3) showed that sjTREC levels declined in an age-dependent manner in bloodstain samples with a correlation coefficient of *r* = −0.8712, which is slightly higher than the *r* = −0.8177 reported by our previous studies. One possible explanation is that the sample size in the present study increased. Another is that the simplified comparative Ct method employed in this study measured the target and endogenous control in samples directly, removing the necessity for determining the absolute target quantity in samples by using standard curve method, in which the quantification accuracy was strongly dependent on the standard curve constructed by a standard dilution series. However, since sjTREC content is a function of thymic output, T cell division and T cell survival, a potential drawback of the proposed age predicting assay could be in the effect of the immune system health status. Besides, as Ferrando-Martínez et al. [19] found, there was a suggestion of an inhomogeneity in thymic function in elderly individuals though our sample number in this age range was small (n = 25 for over 55 years), which might compromise the accuracy of this method. One possible solution for improving the predicting accuracy is to further study the potential impact of environmental (storage time, temperature, and UV radiation etc.), genetic and disease (e.g. lymphocytic choriomeningitis virus, HIV, Epstein-Barr, or cytomegalovirus infections) factors. The other is to combine with measurements of other age indicators independent of thymic function, such as TRF length, mtDNA 4,977 bp fragment deletion, or epigenetic predictors newly reported in recent research [20]. Furthermore, as another strong peripheral thymic function related marker, the relationship of sj/â-TREC ratio [21], [22] and individual age needs further investigation.

To examine influence of the storage time on the blood samples used in our method, we analyzed the specimens of the same individuals after series of storage time. Remarkably, the sjTREC quantification results indicated no statistically significant difference between the fresh DNA samples and those over a 4-week storage time, suggesting the age-estimation formula described in our study was supposed to be available for fresh bloodstains within 4 weeks, which are the most common biological evidence in forensic practice. However, notable loss (0.16–1.93 dCt) in sjTREC contents was detected after 1.5 years of storage in 31 samples. To our knowledge, the dCt (Ct_TBP_-Ct_sjTREC_) loss in stored samples might be due to the different degradation rate between these two DNA targets. As a single chained DNA circle, sjTREC is expected to be more vulnerable to environmental factors than the TBP normalizer, which remains part of the double chained genomic DNA. Then, preliminary sjTREC quantification in old bloodstains was performed using samples routinely stored for 3, 6, 12 and over 20 years respectively in lab conditions. The results showed that though the sjTREC contents were all detectable in these collected old bloodstain samples and highly correlated with donor age, there exists a time-dependent decrease in the correlation coefficient *r*, suggesting the predicting accuracy of this described assay would be deteriorated in aged samples. Therefore, further investigation is required to establish age predicting mode for stored samples, in which a larger number of samples with known storage time span should be involved.

In summary, as an age-related DNA maker, sjTREC quantification is revealed to be suitable for age estimating of bloodstain samples with considerable predicting accuracy and stability within a certain period of storage time. Although complementary analyses are needed before the application in practice, our studies present an established method to evaluate immunological age of a bloodstain. Therefore, as the Study Group on Forensic Age Diagnostics has recommended [23], the future age diagnostics of forensic samples without enough morphologic information should be made with assistance of multiple morphologically independent age-related features, to obtain age estimates as precisely as possible.
